# Usefulness of cardiac magnetic resonance images for prediction of sudden cardiac arrest in patients with mitral valve prolapse: a multicenter retrospective cohort study

**DOI:** 10.1186/s12872-021-02362-2

**Published:** 2021-11-17

**Authors:** Jae-Hyuk Lee, Jae-Sun Uhm, Young Joo Suh, Min Kim, In-Soo Kim, Moo-Nyun Jin, Min Soo Cho, Hee Tae Yu, Tae-Hoon Kim, Yoo Jin Hong, Hye-Jeong Lee, Chi Young Shim, Young Jin Kim, Jun Kim, Jong-Youn Kim, Boyoung Joung, Geu-Ru Hong, Hui-Nam Pak, Gi-Byoung Nam, Kee-Joon Choi, You-Ho Kim, Moon-Hyoung Lee

**Affiliations:** 1 Departments of Cardiology, Severance Cardiovascular Hospital, College of Medicine, Yonsei University, 50-1 Yonsei-ro Seodaemun-gu, Seoul, 03722 Republic of Korea; 2 Departments of Cardiology and Radiology, Severance Hospital, College of Medicine, Yonsei University, Seoul, Republic of Korea; 3 Department of Cardiology, Asan Medical Center, University of Ulsan College of Medicine, Seoul, Republic of Korea; 4 Department of Cardiology, Gangnam Severance Hospital, College of Medicine, Yonsei University, Seoul, Republic of Korea

**Keywords:** Arrhythmia, Gadolinium, Magnetic resonance imaging, Mitral valve prolapse, Sudden cardiac death, Ventricular tachycardia, Ventricular fibrillation

## Abstract

**Background:**

An association has been identified between mitral valve prolapse (MVP) and sudden cardiac arrest (SCA), and ventricular arrhythmias (VA). This study aimed to elucidate predictive factors for SCA or VA in MVP patients.

**Methods:**

MVP patients who underwent cardiac magnetic resonance (CMR) were retrospectively included. Patients with other structural heart disease or causes of aborted SCA were excluded. Clinical characteristics (sex, age, body mass index, histories of diabetes, hypertension, and dyslipidemia) and electrocardiographic (PR interval, QRS duration, corrected QT interval, inverted T wave in the inferior leads, bundle branch block, and atrial fibrillation), echocardiographic [mitral regurgitation grade, prolapsing mitral leaflet, and right ventricular systolic pressure (RVSP)], and CMR [left atrial volume index, both ventricular ejection fractions, both ventricular end-diastolic and systolic volume indexes, prolapse distance, mitral annular disjunction, systolic curling motion, presence of late gadolinium enhancement (LGE), LGE volume and proportion] parameters were analyzed.

**Results:**

Of the 85 patients [age, 54.0 (41.0–65.0) years; 46 men], seven experienced SCA or VA. Younger age and wide QRS complex were observed more often in the SCA/VA group than in the no-SCA/VA group. The SCA/VA group exhibited lower RVSP, more systolic curling motion and LGE, greater LGE volume, and higher LGE proportion. The presence of LGE [hazard ratio (HR), 19.8; 95% confidence interval (CI) 2.65–148.15; *P* = 0.004], LGE volume (HR 1.08; 95% CI 1.02–1.14; P = 0.006) and LGE proportion (HR 1.32; 95% CI 1.08–1.60; P = 0.006) were independently associated with higher risk of SCA or VA in MVP patients together with systolic curling motion in each model.

**Conclusions:**

The presence of systolic curling motion, high LGE volume and proportion, and the presence of LGE on CMR were independent predictive factors for SCA or VA in MVP patients.

## Background

Mitral valve prolapse (MVP), a common valvular heart disease affects 2–3% of the general population [1, 2]. Although MVP is generally regarded as a benign condition, a number of studies have reported an association between MVP and sudden cardiac arrest (SCA) [3–6]. A meta-analysis reported the overall prevalence of SCA in MVP as 217 events per 100,000 person-years, a value noticeably higher than that of the general population, which was recently reported as 42–53 events per 100,000 person-years [5, 7]. The pathogenesis of SCA in MVP remains unclear. Previous studies have proposed several possible mechanisms, including myocardial fibrosis identified on cardiac magnetic resonance (CMR) images, mitral apparatus morphology, and electrophysiological characteristics such as ventricular ectopy burden [6]. The growing interest in risk factors or predictors of SCA is attributable to its considerable occurrence in patients with MVP.

CMR images can be used to analyze the myocardial composition and identify, for example, myocardial fibrosis. Several studies have reported that late gadolinium enhancement (LGE) distribution on CMR is associated with ventricular arrhythmia (VA) [3, 8–10]. Recently, morphological and functional information from CMR images, including mitral annular disjunction (MAD), prolapse distance, and systolic curling, was reported as factors associated with VA in MVP patients [9, 11, 12]. Although CMR images provide useful information about risk for arrhythmia in MVP patients, the guidelines do not recommend CMR as a first-line tool for risk stratification in MVP patients [13].

In this multicenter retrospective study, we aimed to evaluate the risk factors for SCA or VA in MVP patients. Furthermore, we aimed to elucidate usefulness of CMR for prediction of SCA or VA in MVP patients.

## Methods

### Study population

This was a multicenter retrospective cohort study. The study design was approved by the institutional review board (IRB) (IRB number: 4-2019-0747 and 2019-1151) and the study was conducted in accordance with the Declaration of Helsinki. The need to obtain informed consent from the patients and the need for review by a critical event committee were waived by the IRB due to the retrospective nature of this study and the absence of patient identification data presented.

Among patients who were diagnosed with MVP on echocardiography, total 117 patients (aged ≥ 18 years) patients who underwent CMR for any reasons from January 2000 to June 2019 in three university hospitals were retrospectively included. The exclusion criteria were as follows: (1) presence of concomitant structural heart disease other than MVP; (2) presence of possible causes of SCA other than MVP; (3) CMR performed after mitral valve surgery; (4) significant (intervention-requiring) coronary artery disease.

### Electrocardiography and echocardiography

In all patients, a 12-lead Electrocardiography (ECG) was performed using standard methods. The ECG of each patient at the time of diagnosing MVP was reviewed, and their ECG parameters [PR interval, QRS duration, QT interval, QT interval corrected using Bazett’s formula (QTc), inverted T wave in the inferior leads, and presence of bundle branch block and atrial fibrillation] were obtained.

Following standard methods, transthoracic echocardiography was performed in all patients. Echocardiography at the initial diagnosis of MVP was analyzed. The criteria for the diagnosis of MVP included an abnormal systolic valve motion of the mitral leaflet into the left atrium (LA) (≥ 2 mm beyond the annulus) on transthoracic echocardiography [14]. The grades of mitral regurgitation (MR) were categorized into mild, moderate, and severe based on Doppler echocardiography following the standard criteria of the American Society of Echocardiography [15]. The prolapsing mitral leaflet and presence of ruptured chordae tendinae were observed from multiple views. The right ventricular (RV) systolic pressure (RVSP) was estimated using the maximal velocity of tricuspid regurgitation and the conventional simplified Bernoulli’s equation.

### CMR imaging

In institution 1, CMR was performed either using 1.5-T scanner (InteraAchieva; Philips Medical Systems, Best, the Netherlands) or 3.0-T scanner (Magnetom Trio; Siemens Medical Solutions, Erlangen, Germany). In institution 2, it was conducted using a 1.5-T system (Achieva; Philips Healthcare, Best, Netherlands) and a 32-channel cardiac coil. In institution 3, 1.5-T systems (Vision 1.5 T and Avanto 1.5 T; Siemens Medical Systems, Erlangen, Germany) were used. The CMR protocol of each institution was previously described in detail by studies conducted in each center [16–19]. ECG-gated cine imaging was performed using a balanced steady-state free precession sequence. LGE imaging was performed 10 min following the administration of gadobutrol (0.2 mmol/kg, Gadovist; Bayer Schering Pharma AG, Berlin, Germany) at 2 mL/s. Data acquisition was synchronized with ECG in the mid-diastolic phase to minimize motion artifacts.

All CMR images were analyzed off-line using a dedicated software program (cvi2, Circle Cardiovascular Imaging, Calgary, Alberta, Canada). The left ventricular (LV) ejection fraction (EF), LA volume index, right ventricular ejection fraction (RVEF), LV and RV end-diastolic (EDV) and systolic (ESV) volume index, valvular prolapse distance, presence of MAD and systolic curling motion, and LGE volume and proportion were all estimated from the CMR images. From short-axis cine images, the LV and RV volumes and EF were measured using a semi-automatic segmentation in the software, and all volume measurements were normalized to the body surface area. The papillary muscles and trabeculations were included in the LV volume. MAD was defined as a separation between the LA-valve junction and the atrial aspect of the LV free wall and systolic curling motion was defined as an unusual systolic motion of the posterior mitral ring on the adjacent myocardium. The length of MAD was measured from the LA wall-mitral valve leaflet junction to the top of the LV wall during end-systole in long-axis cine images [9, 20]. Prolapse distance and the presence of MAD and systolic curling motion were evaluated from 3 chamber long-axis cine images. LGE represents a relative excess of gadolinium in the pathological tissue compared to the healthy tissue. First, the presence of LGE was visually determined; when LGE was found, its pattern was evaluated. In the short-axis LGE images, the endocardial and epicardial borders of the LV were manually drawn and the volume and extent of LGE (%) were automatically quantified by adopting the 5-standard deviation method. LGE volume was calculated by multiplying the LGE area by section thickness which was obtained by hyperenhancing pixels on the CMR images with manual tracing. LGE proportion was calculated by dividing LGE volume by the LV myocardium, with the resulting quotient multiplied by 100. To reduce effects from imaging artifacts and other confounders, LGE confined to the RV insertion site was ignored [21, 22].

### Grouping of patients and analyses

Medical records, ECG, echocardiography at the time of diagnosing MVP and CMR images were reviewed. Clinical characteristics [sex, age, body mass index (BMI), medical histories of diabetes, hypertension, family history of SCA, and dyslipidemia] were acquired from the medical records. SCA was defined as abrupt cessation of cardiac function resulting in loss of effective circulation when witnessed or within 24 h from being last seen in healthy state when unwitnessed. The VA included ventricular fibrillation and sustained or non-sustained ventricular tachycardia (NSVT) on a single- or 12-lead ECG, Holter monitoring, or treadmill test. We confirmed SCA and VA using medical claims and records retrospectively. Patients were classified based on the presentation of SCA or VA into two categories, as follows: the SCA/VA group and no-SCA/VA group. Baseline characteristics, ECG (PR interval, QRS duration, QTc, inverted T wave in the inferior leads, and the presence of atrial fibrillation and bundle branch block), echocardiographic (MR grade, prolapsed mitral leaflet, presence of ruptured chordae tendinae, and RVSP), and CMR (LVEF, LA volume index, RVEF, LVEDV index, LVESV index, RVEDV index, RVESV index, prolapse distance, MAD, systolic curling motion, presence of LGE, and LGE volume and proportion) findings were compared between the groups. Associations of baseline characteristics and ECG, echocardiographic, and CMR findings with SCA or VA were analyzed.

### Statistical analysis

Baseline characteristics were analyzed using descriptive statistics. Continuous variables were presented as median with inter-quartile range for non-normally distributed variables, whereas categorical variables were presented as frequency and percentages. Continuous and categorical variables were compared using Wilcoxon rank-sum test and Fisher’s exact test, respectively. A Cox regression analysis was used to identify the predictors of SCA or VA in the MVP patients and estimate the hazard ratios (HRs), 95% confidence intervals (CIs), and p-values. The variables selected for the multivariable analysis were those with a p-value of < 0.05 in the univariable analysis. When there was multicollinearity among variables, we used the more significant variable for adjustment in the multivariable Cox regression analysis. LGE-related parameters, including presence of LGE, LGE volume, and LGE proportion, were separately analyzed in three different models because there was multicollinearity among the three variables. Statistical Package for the Social Sciences version 25.0 for Windows (IBM Corporation, Armonk, NY, USA) and R software version 3.6.2 (The R foundation for Statistical Computing, Vienna, Austria) were employed in the analysis of data.

## Results

### Study population and ECG

A total of 117 MVP patients [age, 57.5 (42.3–67.0) years; 53 males] who underwent CMR were screened. Thirty-two patients were excluded due to congenital heart disease, ischemic cardiomyopathy and prior mitral valve surgery. Following this, 85 patients [age, 54.0 (41.0–65.0) years; 46 males] were finally included; of whom, SCA occurred in 5 patients, sustained ventricular tachycardia occurred during treadmill test in 1 patient, and NSVT occurred during treadmill test and Holter monitoring in 1 patient during the 7.2 (3.9–9.1) years’ follow-up.

Baseline characteristics of patients in the SCA/VA and no-SCA/VA groups are presented in Table 1. Patients in the SCA/VA group were younger and had wider QRS complex than those in the no-SCA/VA group. No significant differences were observed between the groups in sex, BMI, family history of SCA/VA, and medical histories of diabetes, and hypertension. The ECG findings revealed no significant differences in the PR interval, QTc, and the presence of inverted T wave in the inferior leads, atrial fibrillation, and right bundle brunch block (RBBB) between the groups.

**Table 1:** Baseline characteristics of MVP patients between two groups

	All subjects (n = 85)	SCA/VA group (n = 7)	No-SCA/VA group (n = 78)	*P*
Age (years)	54.0 (41.0–65.0)	41.0 (33.0–49.0)	55.0 (42.0–65.0)	0.043
Male sex	46 (54.1)	6 (85.7)	40 (51.3)	0.175
Body mass index (kg/m^2^)	22.7 (20.9–25.0)	22.6 (22.2–28.0)	22.7 (20.9–24.9)	0.332
Diabetes mellitus	7 (8.2)	1 (14.3)	6 (7.7)	0.465
Hypertension	22 (25.9)	1 (14.3)	21 (26.9)	0.671
CAD	4 (4.7)	0 (0)	4 (5.1)	> 0.999
Dyslipidemia	8 (9.4)	0 (0)	8 (10.3)	0.830
Family history of SCA	4 (4.7)	0 (0)	4 (5.1)	> 0.999
*ECG*				
PR interval (ms)	164.0 (148.0–186.0)	181.0 (166.0–198.0)	160.0 (144.0–186.0)	0.149
QRS duration (ms)	96.0 (88.0–106.0)	114.0 (104.0–127.0)	96.0 (88.0–104.0)	0.007
QTc (ms)	444.0 (426.0–462.0)	437.0 (421.5–458.5)	444.0 (426.0–462.0)	0.689
Inverted T wave in inferior leads	11 (12.9)	2 (28.6)	9 (11.5)	0.485
Atrial fibrillation	25 (29.4)	2 (28.6)	23 (29.5)	> 0.999
LBBB	1 (1.2)	0 (0)	1 (1.3)	> 0.999
RBBB	6 (7.1)	1 (14.3)	5 (6.4)	0.993

Values are presented as number (%) and median (first and third quartiles) for categorical, and continuous variables, respectively

*ECG* Electrocardiography, *LBBB* left bundle branch block, *MVP* mitral valve prolapse, *QTc* QT interval corrected by Bazett’s formula, *RBBB* right bundle branch block, *SCA* sudden cardiac arrest, *VA* ventricular arrhythmia

### Imaging findings

Echocardiographic and CMR findings of patients in the SCA/VA and no-SCA/VA groups are presented in Table 2. The time interval between diagnosis of MVP and CMR was 0 (0–7) months. Lower RVSP was observed in the SCA/VA group than in the no-SCA/VA group, as demonstrated by echocardiographic findings. However, no significant differences were observed in the MR grade, involved leaflet, and the presence of ruptured chordae tendinae between the groups. Among CMR findings, the presence of systolic curling motion and LGE, and LGE volume and proportion were significantly greater in the SCA/VA group than in the no-SCA/VA group. However, no significant differences were observed in the LVEF, RVEF, LVEDV index, LVESV index, RVEDV index, and RVESV index between the groups.

**Table 2:** Imaging findings of MVP patients between two groups

	All subjects (n = 85)	SCA/VA group (n = 7)	No-SCA/VA group (n = 78)	*P*
*Echocardiography*				
MR grade				0.153
No	4 (4.7)	1 (14.3)	3 (3.8)	
Mild	9 (10.6)	2 (28.6)	7 (9.0)	
Moderate	15 (17.6)	0 (0.0)	15 (19.2)	
Severe	57 (67.1)	4 (57.1)	53 (67.9)	
Involved leaflet				0.503
Anterior	48 (56.5)	5 (71.4)	43 (55.1)	
Posterior	29 (34.1)	1 (14.3)	28 (35.9)	
Both	8 (9.4)	1 (14.3)	7 (9.0)	
RVSP (mmHg)	34.0 (28.0–47.0)	23.0 (21.0–27.0)	35.0 (28.0–51.0)	0.002
Ruptured chordae tendinae	26 (30.6)	1 (14.3)	25 (32.1)	0.583
*CMR*				
LVEF (%)	60.0 (52.0–68.0)	51.0 (49.0–66.5)	60.0 (52.0–68.0)	0.522
LA volume index	56.5 (35.1–79.7)	58.2 (40.0–80.0)	30.0 (29.5–60.2)	0.111
LVEDV index (mL/m^2^)	122.3 (99.4–155.1)	111.6 (83.1–145.3)	122.5 (100.3–159.5)	0.480
LVESV index (mL/m^2^)	50.1 (32.4–72.8)	54.9 (23.1–74.2)	49.6 (33.3–72.7)	0.716
RVEF (%)	53.0 (43.0–60.0)	55.0 (50.0–60.0)	53.0 (41.0–59.0)	0.437
RVEDV index (mL/m^2^)	90.5 (69.3–129.0)	96.3 (74.0–118.5)	90.5 (69.3–128.7)	0.936
RVESV index (mL/m^2^)	40.5 (29.8–68.1)	39.2 (28.2–57.8)	41.3 (30.5–73.4)	0.762
Prolapse distance (mm)	6.6 (4.1–8.4)	8.0 (4.8–9.7)	6.6 (3.9–8.4)	0.274
Mitral annular disjunction	13 (15.7)	3 (42.9)	10 (13.2)	0.127
Systolic curling motion	9 (10.8)	3 (42.9)	6 (7.9)	0.027
Presence of LGE	8 (9.4)	4 (57.1)	4 (5.1)	< 0.001
LGE volume (g)	0 (0–0)	1.3 (0.0–15.1)	0 (0–0)	< 0.001
LGE proportion (%)	0 (0–0)	1.2 (0.0–7.8)	0 (0–0)	< 0.001

Values are presented as number (%) and median (first and third quartiles) for categorical, and continuous variables, respectively

*CMR* Cardiac magnetic resonance, *LA* left atrial, *LGE* late gadolinium enhancement, *LVEDV* left ventricular end diastolic volume, *LVEF* left ventricular ejection fraction, *LVESV* left ventricular end systolic volume, *MR* mitral regurgitation, *MVP* mitral valve prolapse, *RVEDV* right ventricular end diastolic volume, *RVEF* right ventricular ejection fraction, *RVESV* right ventricular end systolic volume, *RVSP* right ventricular systolic pressure, *SCA* sudden cardiac arrest, *VA* ventricular arrhythmia

**Fig. 1:**
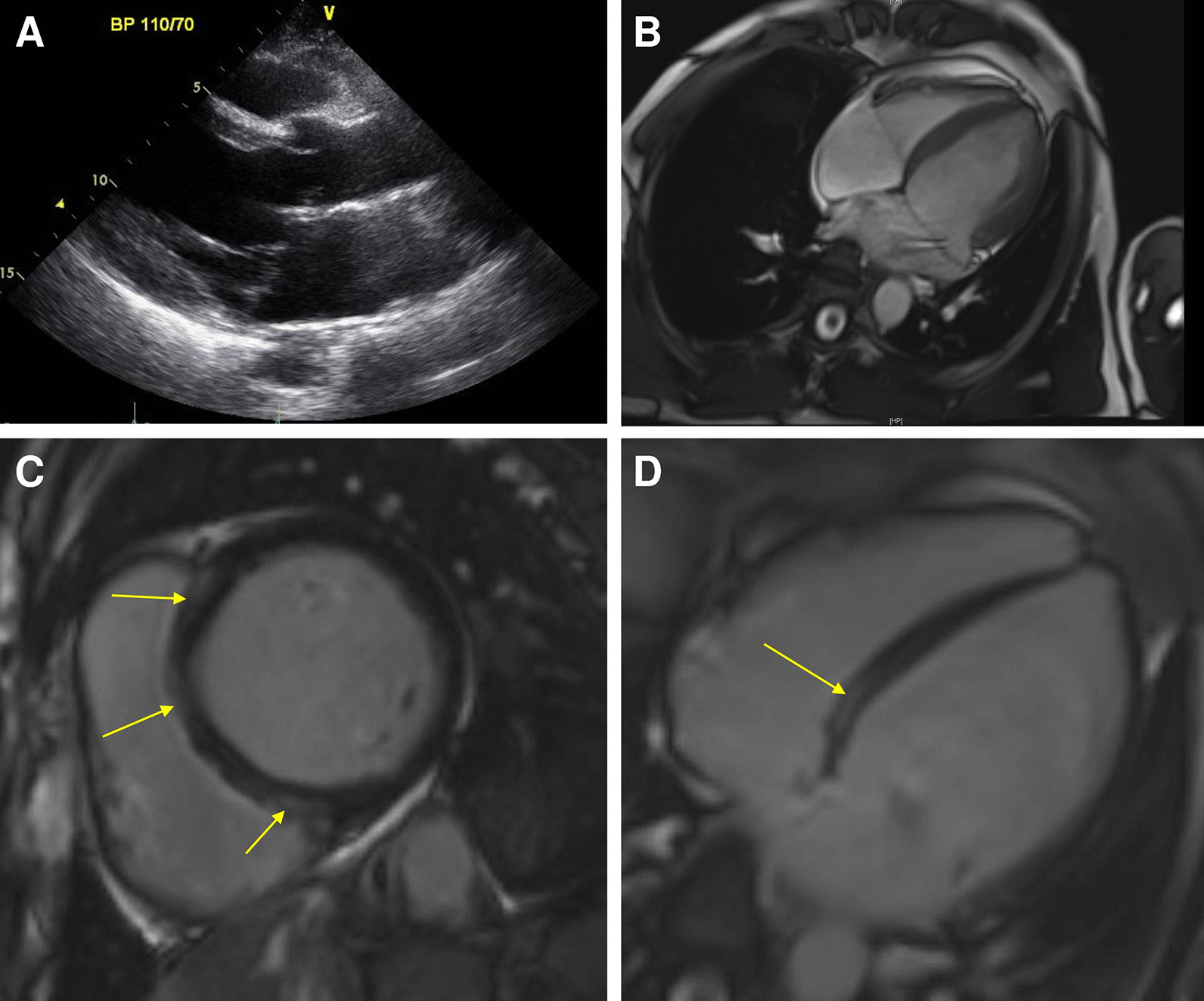
Echocardiographic and CMR findings of an MVP patient who experienced SCA. Echocardiographic image of the parasternal long axis view (**A**) and CMR image of 4-chamber view (**B**) showing prolapse of both mitral leaflets. CMR images of the short axis view (**C**) and 4-chamber view (**D**) showing LGE (arrows) at the mid-layer of the basal septum. *CMR* cardiac magnetic resonance, *LGE* late gadolinium enhancement, *MVP* mitral valve prolapse, *SCA* sudden cardiac arrest

Brief characteristics and LGE findings of seven patients who experienced SCA or VA and of four patients in whom LGE was identified on CMR without SCA or VA are presented in Table 3. In patients in the SCA/VA group, the LGE was mostly located in the inferior and inferolateral segments of the basal LV or papillary muscles of the LV. However, some patients had multifocal locations of LGE (Table 3). Examples of echocardiographic and CMR images of a MVP patient who experienced SCA are shown in Fig. 1.

**Table 3:** Clinical and CMR findings of patients in SCA/VA group and in no SCA/VA group with presence of LGE

Range of age	Sex	SCA/VA type	ECG at event	LGE on CMR	LGE locations and patterns
*SCA/VA group*					
30’s–40’s	Male	SCA	VF	Yes	Multifocal with patchy pattern: mesocardial at the basal to mid anteroseptal segment of LV; mesocardial to transmural at the basal to mid anterior, inferolateral, and inferior segments of LV
40’s–50’s	Male	SCA	VF	Yes	Mesocardial at the basal inferoseptal segment of LV
20’s–30’s	Male	SCA	VF	Yes	Multifocal: transmural at the basal inferior segment of LV; subendocardial at the basal anterolateral and inferolateral segments of LV
40’s–50’s	Male	SCA	VF	Yes	Anterolateral and posteromedial papillary muscles of LV
10’s–20’s	Male	SCA	VF	No	–
60’s–70’s	Female	Sustained VT during treadmill test	VT	No	–
50’s–60’s	Male	NSVT on Holter monitoring/NSVT during treadmill test	NSVT	No	–
*No-SCA/VA group*					
60’s–70’s	Female	–	–	Yes	Focal anterolateral papillary muscle
50’s–60’s	Male	–	–	Yes	Basal septum of LV with linear pattern
50’s–60’s	Male	–	–	Yes	Basal septum mid layer of LV with linear pattern
70’s–80’s	Male	–	–	Yes	Focal distal portion of posterior papillary muscle

*CMR* Cardiac magnetic resonance, *ECG* electrocardiography, *LGE* late gadolinium enhancement, *LV* left ventricle, *NSVT* non-sustained ventricular tachycardia, *SCA* sudden cardiac arrest, *VA* ventricular arrhythmia, *VF* ventricular fibrillation, *VT* ventricular tachycardia

### Risk factors for SCA/VA

In model 1, the QRS duration (HR 1.08 [1.01–1.14], *P* = 0.024), presence of systolic curling motion (HR 13.04 [1.37–124.45], *P* = 0.026), and presence of LGE (HR 19.8 [2.65–148.15], *P* = 0.004) were independently associated with SCA or VA (Table 4). LGE volume (HR 1.08 [1.02–1.14], *P* = 0.006) and LGE proportion (HR 1.32 [1.08–1.60], *P* = 0.006) were independently associated with SCA or VA in model 2 and model 3, respectively. QRS duration and systolic curling motion were consistently associated with SCA or VA (Table 4).

**Table 4:** Cox regression analysis for SCA or VA in patients with MVP

	Univariable		Multivariable (Model 1)		Multivariable (Model 2)		Multivariable (Model 3)	
	HR (95% CI)	*P*	HR (95% CI)	*P*	HR (95% CI)	*P*	HR (95% CI)	*P*
Male sex	6.28 (0.75–52.28)	0.089						
Age (year)	0.96 (0.91–10.00)	0.050	1.07 (0.98–1.17)	0.150	1.10 (0.99–1.23)	0.076	1.12 (0.99–1.25)	0.063
BMI (kg/m^2^)	1.14 (0.98–1.33)	0.089						
Diabetes mellitus	1.81 (0.22–15.1)	0.584						
Hypertension	0.43 (0.05–3.54)	0.429						
Dyslipidemia	0.04 (0–1453.85)	0.550						
*ECG*								
PR interval (ms)	1.01 (0.99–1.03)	0.199						
QRS duration (ms)	1.05 (1.02–1.08)	0.003	1.08 (1.01–1.14)	0.024	1.06 (1.01–1.12)	0.020	1.07 (1.01–1.14)	0.023
QTc (ms)	0.99 (0.97–1.02)	0.645						
Atrial fibrillation	0.85 (0.16–4.38)	0.845						
BBB	2.00 (0.21–19.45)	0.550						
Inverted T wave in inferior leads	2.64 (0.51–13.67)	0.248						
*Echocardiography*								
MR grade								
Not severe	[Ref]	[Ref]						
Severe	0.68 (0.15–3.09)	0.618						
Involved leaflet								
One leaflet	[Ref]	[Ref]						
Both leaflet	2.31 (0.27–19.89)	0.445						
Ruptured chordae tendinae	0.36 (0.04–2.99)	0.342						
RVSP (mmHg)	0.85 (0.74–0.97)	0.015	0.89 (0.77–1.03)	0.113	0.84 (0.68–1.05)	0.123	0.86 (0.69–1.06)	0.148
*CMR*								
LVEF	0.99 (0.94–1.05)	0.748						
LVEDV index	0.99 (0.98–1.01)	0.523						
LVESV index	1.00 (0.98–1.02)	0.806						
RVEF	1.02 (0.96–1.08)	0.503						
RVEDV index	1.00 (0.98–1.02)	0.769						
RVESV index	0.99 (0.96–1.02)	0.601						
LA volume index	0.97 (0.94–1.01)	0.112						
Prolapse distance	1.01 (0.91–1.11)	0.916						
MAD^*^	5.64 (1.23–25.81)	0.026						
Systolic curling motion^*^	11.17 (2.23–55.89)	0.003	13.04 (1.37–124.45)	0.026	55.23 (2.18–1402.07)	0.015	110.9 (2.93–4191.92)	0.011
Presence of LGE^†^	14.48 (3.19–65.68)	0.001	19.8 (2.65–148.15)	0.004	–	–	–	–
LGE volume (g)^†^	1.06 (1.03–1.10)	< 0.001	–	–	1.08 (1.02–1.14)	0.006	–	–
LGE proportion (%)^†^	1.22 (1.10–1.34)	< 0.001	–	–	–	–	1.32 (1.08–1.60)	0.006

*BBB* Bundle branch block, *BMI* body mass index, calculated as weight in kilograms divided by the square of height in meters, *CMR* cardiac magnetic resonance, *ECG* electrocardiography, *HR* hazard ratio, *LGE* late gadolinium enhancement, *LVEDV* left ventricular end diastolic volume, *LVEF* left ventricular ejection fraction, *LVESV* left ventricular end systolic volume, *MAD* mitral annular disjunction, *MR* mitral regurgitation, *MVP* mitral valve prolapse, *Ref* reference, *RVEDV* right ventricular end diastolic volume, *RVEF* right ventricular ejection fraction, *RVESV* right ventricular end systolic volume, *RVSP* right ventricular systolic pressure, *SCA* sudden cardiac arrest, *VA* ventricular arrhythmia

^*^Only systolic curling motion was used on the multivariable Cox regression analysis due to multicollinearity with MAD

^†^These three variables were analyzed separately in Model 1, Model 2, and Model 3 due to milticollinearity

## Discussion

### Main findings

The following are the main findings of this study: (1) MVP patients who experienced SCA or VA were younger and had wider QRS complex, lower RVSP, more frequent systolic curling motion and LGE on CMR, greater LGE volume, and higher LGE proportion than those who did not experience SCA or VA, and (2) the presence of LGE, high LGE volume and proportion, and systolic curling motion on CMR were independently associated with SCA or VA in MVP patients.

### Previous studies on risk factors for SCA in MVP patients

Previous studies have reported that young age, female sex, severe valve dysfunction, bileaflet MVP, ventricular ectopy, and LGE on CMR are possible risk factors for SCA in patients with MVP [3–6, 23]. In the present study, age, female sex, MR grade, and involved leaflet were not found to be associated with SCA or VA in MVP patients. Although there are many suggested predictive parameters for SCA in MVP patients, these vary and are inconsistent among studies. This may be because the inclusion criteria differed among studies, and the obtained clinical, laboratory, and imaging measurements were different. The adoption of different definitions for arrhythmic events among studies may be and additional explanation. Because of these inconsistent results, there is still no standard consensus regarding risk stratification for SCA in MVP patients.

### Significance of LGE on CMR as a risk factor for SCA in patients with MVP

LGE on CMR may indicate focal myocardial fibrosis, which can be a substrate of VA [24, 25]. There is an established association between the presence of LGE and SCA. However, most studies that aimed to clarify the association between LGE and SCA investigated ischemia-related disease [25–27]. A previous study reported that LGE could be a promising marker for the prediction of SCA even in patients with MVP [3]. In line with the previous studies, the present study demonstrated that the presence of LGE was associated with SCA. The previous studies have also discussed that the patterns and locations of LGE in MVP patients who experienced SCA were located in the basal infero-lateral wall and papillary muscles [3, 10]. In the present study, the LGE patterns in these patients had an inconsistent LGE distribution with that identified in previous studies. Most patients in the present study had moderate to severe MR, and most of them underwent CMR immediately before the operation for MVP. Therefore, many patients in this study may already have had chronic remodeling of LV, which could have caused the different, and even severer, LGE patterns compared with those as previously reported.

Several attempts have been conducted regarding the prediction of SCA or VA with CMR with advancements in CMR technology. In a previous study, the extent of LGE was reported as a strong predictor of recurrent adverse events among SCA survivors [27]. This concept was employed in the MVP patients in this study. The results of the present study revealed that LGE volume and proportion were higher in the SCA group than in the no-SCA group and were independently associated with SCA or VA. There is a paucity of the data about LGE volume and proportion as predictive markers of SCA or VA in patients with MVP. Recently, morphofunctional parameters, such as MAD or systolic curling motion, were reported as predictive markers for SCA in MVP [4, 28–30]. In the present study, systolic curling motion, which is usually shown together with MAD, was reported as an independent predictor for SCA or VA in MVP patients. This finding could also support the usefulness of CMR for assuming high risk of SCA in patients with MVP. Further prospective studies with a large number of patients are warranted.

### ECG and echocardiographic findings

Past studies have reported a relationship between T wave inversion in the inferior leads, RBBB, and frequent ventricular ectopy, and SCA risk in MVP patients [3, 31]. The present study revealed that T wave inversion in the inferior leads and bundle branch block were not associated with SCA or VA in MVP patients. The association between a wide QRS complex and sudden cardiac death in the general population were previously reported [32]. However, no studies have reported an association between the QRS duration and SCA in MVP patients. In the present study, the results showed that a wide QRS complex was independently associated with SCA or VA even in MVP patients.

A probable association has been proposed between the severity of MR, myxomatous degeneration of the mitral valve leaflets, involvement of both leaflets, and ruptured chordae tendinae and SCA. In the present study, the severity of MR and involvement of both leaflets were not statistically significant. Since this study was conducted in tertiary medical centers, biased subjects, together with small number of events, could be a reason of different results compared to previous study.

### Study limitations

There were several limitations to this study. First, as it was a retrospective cohort study, there could be some missed variables in this study which were previously reported as possible predictors for SCA/VA. Furthermore, since data collection and evaluation depended on the medical records and imaging review, there were some limitations to obtain clear information about the situation of patients. Second, the numbers of the patients and event numbers were small. That could be a cause of statistical limitations to analyze risk factors. In some variables that showed no statistical significance, type II error might occur due to the small number of patients. Third, selection bias might be involved in this study design. As this study was conducted in tertiary medical centers, MVP was advanced in the most patients. Moreover, since this study included the only MVP patients who had undergone CMR, these patients would represent a higher risk subset. This biased selection could be a reason for the relatively higher prevalence of SCA or VA (1,143 events per 100,000 person-years) reported in this study than in the general population. Fourth, the LGE analysis was performed on CMR images obtained using 1.5 T and 3.0 T scanners; differences in resolution could have compromised LGE quantification. Accordingly, analysis of LGE was performed as core lab analysis by one radiologist. Fifth, in this study, NSVT was classified alongside SCA. The association between episodes of NSVT and SCA remains unclear. However, it has been reported that patients with MVP and NSVT were more likely to have inducible sustained ventricular tachycardia with programmed stimulation compared with a structurally normal heart [33]. In addition, since Holter monitoring and treadmill test were performed in 7 and 6 patients in the SCA/VA group and 23 and 10 patients in the no-SCA/VA group, respectively, there could be undetected NSVT. Further prospective large-scale studies are therefore necessary.

## Conclusions

The presence of systolic curling motion, high LGE volume and proportion, and the presence of LGE on CMR were identified as independent predictive factors for SCA or VA in MVP patients.

## Data Availability

The datasets used and/or analysed during the current study are available from the corresponding author on reasonable request.

## Abbreviations

BMI: Body mass index
CI: Confidence interval
CMR: Cardiac magnetic resonance
ECG: Electrocardiography
EDV: End-diastolic volume
EF: Ejection fraction
ESV: End-systolic volume
HR: Hazard ration
IRB: Institutional review board
LA: Left atrium or left atrial
LGE: Late gadolinium enhancement
LV: Left ventricular
MAD: Mitral annular disjunction
MR: Mitral regurgitation
MVP: Mitral valve prolapse
NSVT: Non-sustained ventricular tachycardia
QTc: QT interval corrected using Bazett’s formula
RBBB: Right bundle brunch block
RV: Right ventricular
RVEF: Right ventricular ejection fraction
RVSP: Right ventricular systolic pressure
SCA: Sudden cardiac arrest
VA: Ventricular arrhythmia
